# Comparison of the Efficacy and Safety of Low Molecular Weight Heparins and Fondaparinux in Patients With COVID-19: A Systematic Review and Meta-Analysis

**DOI:** 10.7759/cureus.69904

**Published:** 2024-09-22

**Authors:** Laith G Shareef, Mustafa M Noori, Aumnia G Shareef, Ali H Mustafa

**Affiliations:** 1 Therapeutics, Al-Rasheed University College, Baghdad, IRQ; 2 Pharmacology, Al-Rasheed University College, Baghdad, IRQ; 3 Pharmacology, Saad Al-Witry Neuroscience Hospital, Baghdad, IRQ; 4 Pharmacology, Medical City, Baghdad, IRQ

**Keywords:** bleeding, covid-19, meta-analysis, mortality, sars-cov-2, venous thromboembolism

## Abstract

Individuals diagnosed with COVID-19 are at a higher risk of arterial and venous thrombosis, mostly pulmonary microvascular thrombosis, which may significantly impair treatment and result in morbidity. This is a systematic review and meta-analysis of research papers that aim to evaluate the risk of bleeding and thrombosis among patients treated with low molecular weight heparin or fondaparinux (LMWH/F). Additionally, we measured the overall death events. This study was conducted in compliance with the Preferred Reporting Items for Systematic Reviews and Meta-Analyses (PRISMA) criteria. A search was conducted in the Clinicaltrials.gov, PubMed, Scopus, and Web of Science databases to identify observational cohort studies and randomized-controlled clinical trials (RCTs) that compared LMWH/F in proven COVID-19 patients. A total of 220 people from two studies were included. Patients who were treated with fondaparinux had a lower risk of developing venous thromboembolism (VTE) (odds ratio (OR) 0.39; 95% confidence interval (CI) (0.14, 1.096); p = 0.168); pulmonary embolism (OR 0.169, 95% CI (0.021, 1.356), p = 0.094); and deep vein thrombosis compared to patients who received LMWH therapy. The data show a lower mortality rate in the LMWH groups (OR 1.135, 95% CI (0.463, 2.785), p = 0.781) and a lower frequency of bleeding (OR 1.657, 95% CI (0.456, 5.908), p = 0.436). Both drugs have shown anti-thrombotic properties in COVID-19 patients. Fondaparinux was somewhat more effective in reducing thrombosis episodes. This research demonstrates the safe use of LMWH for VTE prophylaxis in hospitalized COVID-19 patients based on bleeding and mortality outcomes.

## Introduction and background

The COVID-19 infectious disease was first detected in December 2019 in Wuhan City, China. It resulted in a worldwide crisis, and because of its resemblance to severe acute respiratory syndrome (SARS), it is often known as SARS-CoV-2 in clinical investigations [1]. The occurrence of coagulation problems is prevalent in COVID-19 and is correlated with the severity of the illness. The occurrence of a viral illness after inflammatory reactions leads to a disruption in procoagulant and anticoagulant processes, mostly influenced by endothelial pathology. Individuals who contract COVID-19 with a prior medical history of coagulation disorders are at greater risk for associated dangers compared to other patients [2].

The primary irregularities in coagulation observed in patients with COVID-19 pneumonia correspond to elevated levels of both fibrinogen and D-dimer, which are frequently accompanied by moderate thrombocytopenia. A larger mortality rate has been linked to elevated D-dimer levels. Prothrombin time (PT) and activated partial thromboplastin time (aPTT) can be unusually brief in a subset of COVID-19 cases [3]. As an acute-phase reaction, aPTT is frequently associated with increased factor VIII. A disseminated intravascular coagulopathy-like condition may appear in more severely affected people, characterized by a relatively mild prolongation of PT and aPTT, whereas fibrinogen typically remains normal or elevated [4].

After considering these findings, global stakeholders and agencies have developed guidelines for the utilization of antithrombotic agents in COVID-19 patients who are admitted to the hospital [5]. The American Society of Hematology advises the implementation of preventive anticoagulation for all nonpregnant hospitalized adults, unless there are specific reasons against it, and they additionally suggest administering therapeutic doses of anticoagulants when there is evidence or a strong suspicion of thrombosis based on clinical assessment. Nevertheless, there is not enough evidence to justify the use of intermediate or high (therapeutic) anticoagulant dosages in situations other than this. Furthermore, the impact on mortality of using no anticoagulation compared to anticoagulation, as well as the effect of various antithrombotic dosage regimens, is still uncertain [6,7].

Reports indicate that direct oral anticoagulants are considered safer than low molecular weight heparin (LMWH) in individuals with COVID-19. However, separate research found no notable variation in the risk of massive bleeding and clinically significant nonmajor bleeding comparing fondaparinux (F) and unfractionated heparin (UFH) [8,9]. We carried out a systematic review and meta-analysis of research papers assessing the risk of bleeding and thrombosis events among COVID-19 patients who had been treated with LMWH/F. Additionally, we measured the overall death events of patients given the above-mentioned medications.

## Review

Methods

Study Design

A meta-analysis of studies was conducted to compare the effectiveness of fondaparinux and low molecular weight heparin in reducing the risk of thrombosis, bleeding, and mortality in COVID-19-infected people. This study complied with the Preferred Reporting Items for Systematic Reviews and Meta-Analyses (PRISMA) criteria [10]. This review has been registered in PROSPERO 2022 with the registration number CRD42022335876.

Eligibility Criteria

After evaluating the suitability of titles and abstracts, two separate researchers selected possibly relevant papers. A set of criteria was implemented to identify studies that were eligible for inclusion: studies that included patients of any age, individuals with a COVID-19 diagnosis that was confirmed by a positive genetic test of any severity, individuals who received LMWH/F at any dose, and published data with results of interest. Additional assessments were excluded from non-English publications, case reports, commentaries, narrative and systematic reviews, and non-peer-reviewed research.

Information Sources

We conducted a search on the following databases and trials registry: Clinicaltrials.gov, PubMed, Scopus, and Web of Science. To identify additional studies, an avalanche search was conducted using Google Scholar to look for and filter papers citing articles suitable for full-text review and by perusing the reference listings of indicated publications. On June 7, 2024, we reviewed the database search, and on June 8, 2024, we revised the snowball and further queries.

Search Strategy

The search criteria were successfully merged using appropriate Boolean operators. Furthermore, the references to the chosen papers were reviewed to confirm the thoroughness of the search. The search methodology used for the research is detailed in Table 1.

**Table 1:** Search terms AND: Articles with both keywords, OR: Articles with either or both keywords

Databases or trials registry	Search terms
Clinicaltrials.gov	COVID-19, “Coronavirus Disease 2019” OR “SARS-CoV-2” OR “severe acute respiratory syndrome coronavirus 2”) AND (“Clinical outcomes” OR “Treatment outcomes” AND (Low molecular weight heparin AND Fondaparinux) AND (“Venous thromboembolism” OR “Deep vein thrombosis” OR “Pulmonary embolism”) AND “Mortality” AND “Bleeding”
PubMed	COVID-19, "Coronavirus Disease 2019" OR "SARS-CoV-2" OR "Severe acute respiratory syndrome coronavirus 2") AND ("Clinical outcomes" OR "Treatment outcomes" AND (Low molecular weight heparin AND Fondaparinux) AND ("Venous thromboembolism" OR "Deep vein thrombosis” OR "Pulmonary embolism") AND "Mortality" AND "Bleeding"
Scopus	COVID-19, "Coronavirus Disease 2019" OR "SARS-CoV-2" OR "Severe acute respiratory syndrome coronavirus 2") AND ("Clinical outcomes" OR "Treatment outcomes" AND (Low molecular weight heparin AND Fondaparinux) AND ("Venous thromboembolism" OR "Deep vein thrombosis” OR "Pulmonary embolism") AND "Mortality" AND "Bleeding"
Web of Science	COVID-19, "Coronavirus Disease 2019" OR "SARS-CoV-2" OR "Severe acute respiratory syndrome coronavirus 2") AND ("Clinical outcomes" OR "Treatment outcomes" AND (Low molecular weight heparin AND Fondaparinux) AND ("Venous thromboembolism" OR "Deep vein thrombosis” OR "Pulmonary embolism") AND "Mortality" AND "Bleeding"

Selection Process

**Figure 1:**
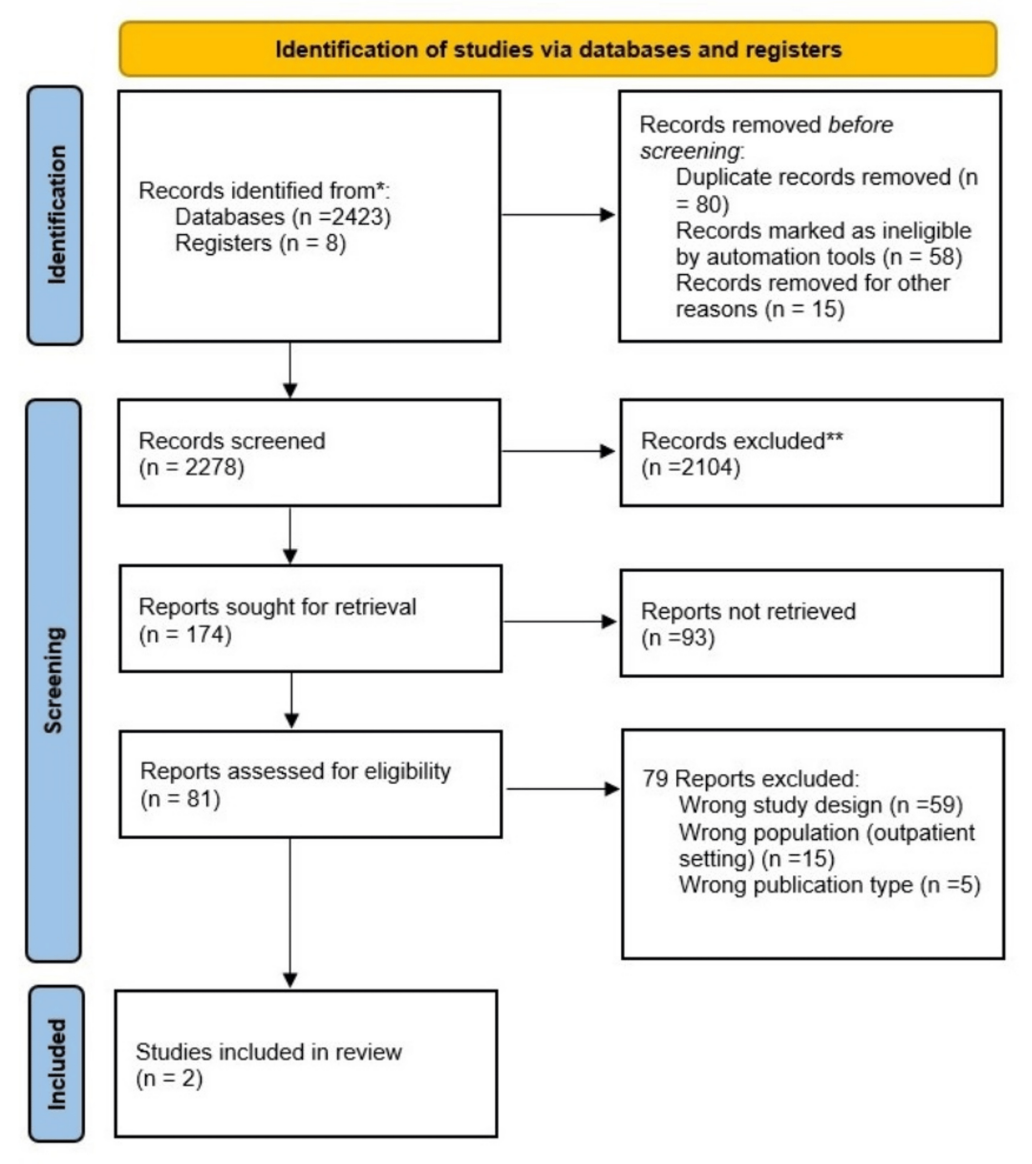
Flow diagram illustrating the criteria used to include papers that were deemed eligible in the meta-analysis * Indicates studies reviewed. ** Indicates studies excluded.

We uploaded all the records found by our search method to Rayyan Intelligent Systematic Review software (Rayyan Systems, Cambridge, MA, USA). Excluded from the list were duplicate articles. Upon reviewing the title and abstract of the first 100 entries, the authors addressed any discrepancies until an agreement was reached. Next, the authors carefully examined the titles and abstracts of all the publications obtained. A definitive consensus on which articles to display in full text was reached via deliberation in the event of disagreement. Additionally, the authors independently assessed whole papers to ascertain their appropriateness for inclusion. In cases of controversy, a consensus on eligibility was achieved via discussion. The search process was recorded in the PRISMA flow chart, which displays the studies that were included and those that were omitted, along with their appropriate arguments, as shown in Figure 1.

Data Collection Process

Analysis of the extracted data was conducted, and any inconsistencies were addressed by discussion. The provided data was imported to Comprehensive Meta-Analysis (CMA) version 3.3.070 (Biostat Inc., Frederick, MD, USA), a computer program designed for meta-analysis.

Study Risk of Bias Assessment

The validity of the data was evaluated by a rigorous assessment utilizing the Joanna Briggs Institute (JBI) meta-analysis for cross-sectional, case-control, cohort studies, and randomized clinical trials. The risk of bias for the observation study using case-control and cohort designs was evaluated based on the following criteria: suitability of the sample frame, suitability of the research participants, sufficient sample size, description of study subjects and setting, rationale for sample size, description of power, variance and effect estimations, valid methods for identifying the condition, measurement of a standard and reliable requirement, suitability of statistical analysis, and satisfactory response rate. The risk assessment criteria were denoted by the binary values 'yes', 'no', 'unclear', or 'not available'. A score of one (1) was assigned to 'yes' and zero (0) to the remainder. The likelihood of bias was deemed low if the overall score exceeded 70%, moderate if it was between 50% and 69%, and high if it was between 0% and 49%. The bias evaluations were conducted separately by two authors.

Data Analysis

The effectiveness and safety of LMWH and fondaparinux in COVID-19 patients were assessed by calculating odds ratios (ORs) with a 95% confidence interval (CI). Statistical significance was indicated by a p-value less than 0.05; this was analyzed statistically using CMA version 3.3.070.

Results

**Figure 2:**
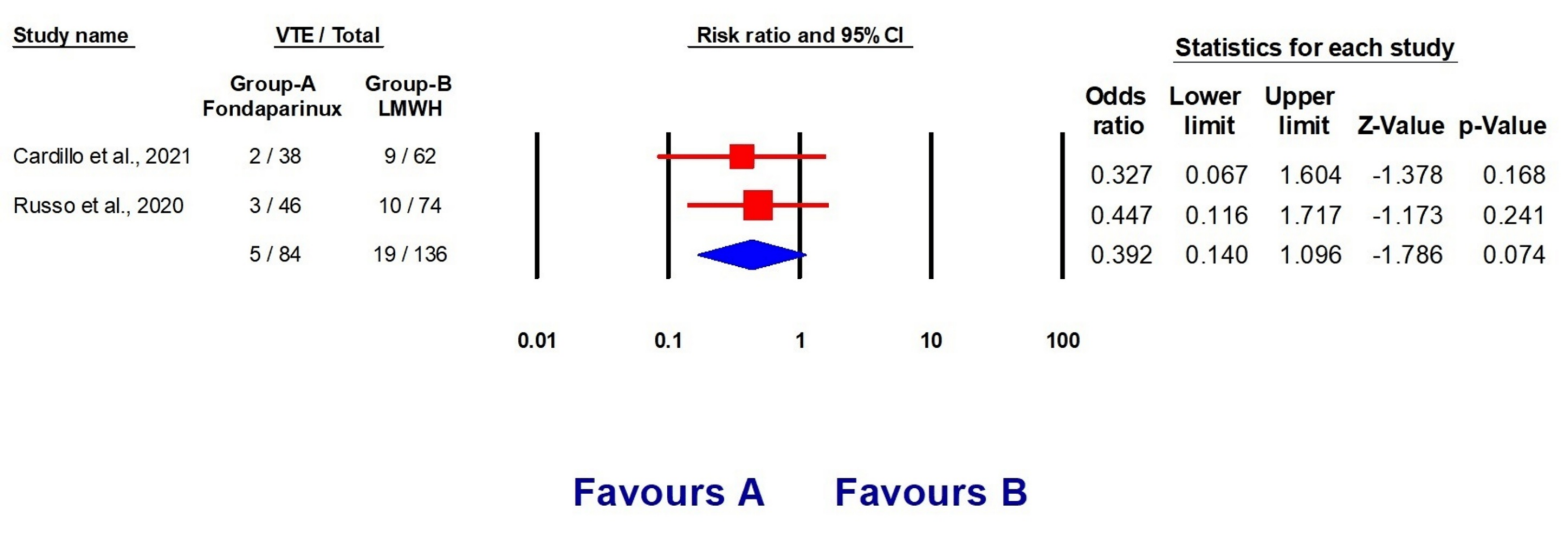
Forest plots explaining the prophylactic effect of fondaparinux and LMWH against VTE development LMWH: Low molecular weight heparin, VTE: Venous thromboembolism

**Figure 3:**
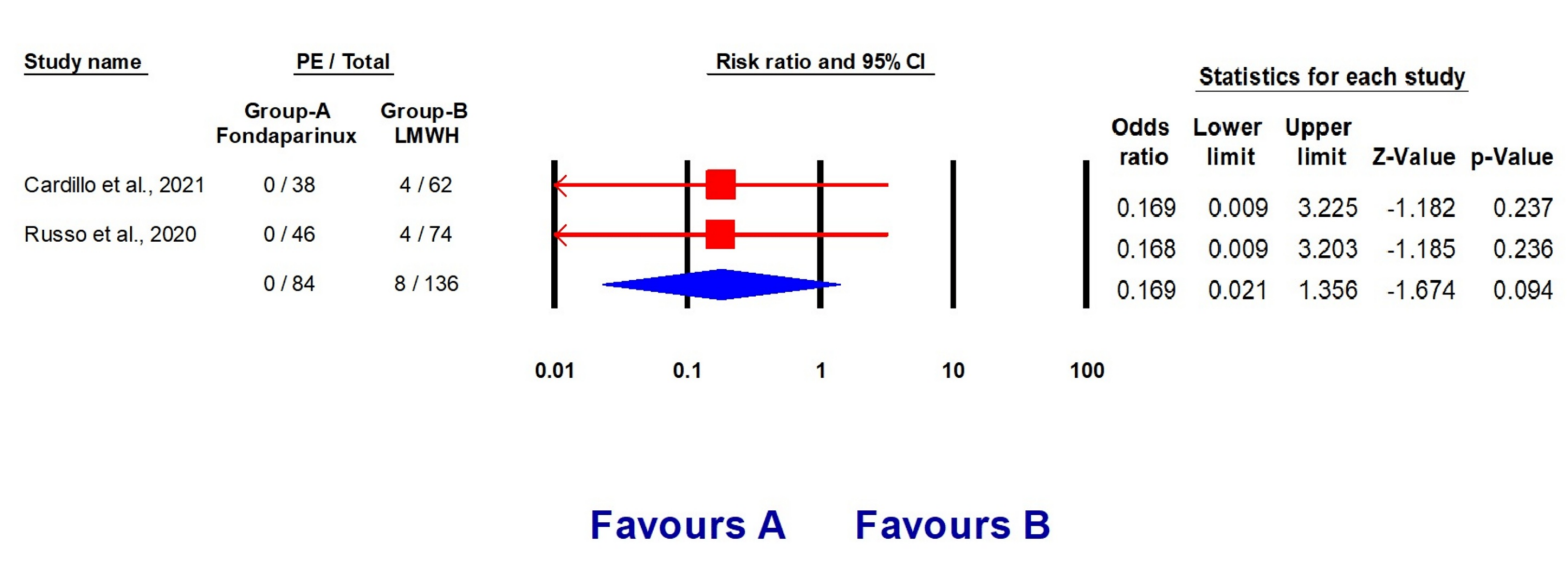
Forest plots explaining the prophylactic effect of fondaparinux and LMWH against PE development LMWH: Low molecular weight heparin, PE: Pulmonary embolism

**Figure 4:**
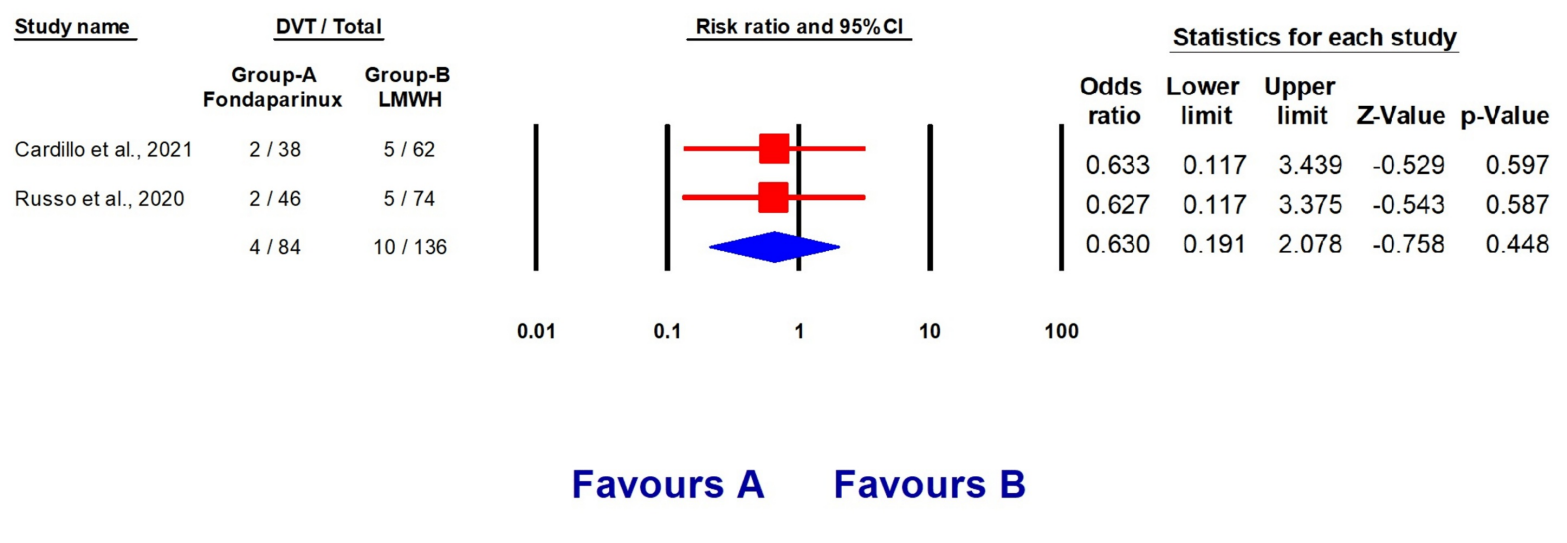
Forest plots explaining the prophylactic effect of fondaparinux and LMWH against DVT development LMWH: Low molecular weight heparin, DVT: Deep venous thromboembolism

Our analysis included two studies [11,12] with a total of 220 individuals. Among them, 127 (57.72%) were males, and 84 (38.18%) were on fondaparinux treatment. The baseline features of the included studies are outlined in Table 2, and the total events are presented in Table 3. Patients who were administered fondaparinux had a reduced likelihood of developing venous thromboembolism (OR 0.39, 95% CI (0.14, 1.0960, p = 0.168), a lower risk of developing pulmonary embolism (OR 0.169, 95% CI (0.021, 1.356), p = 0.094), and a lower risk of developing deep vein thrombosis compared to patients who received LMWH therapy (Figures 2-4).

**Table 2:** Characteristics of included studies F: Fondaparinux, LMWH: Low molecular weight heparin

Author and year of publication	Total no. of patients	Patients on LMWH	Patients on F	LMWH dose	F dose	Design	The median follow-up (days)
Cardillo et al., (2021) [11]	100	62	38	4000 – 6000 IU/day	2.5 mg/ day	Retrospective	28
Russo et al., (2020) [12]	120	74	46	4000 – 6000 IU/day	2.5 mg/ day	Retrospective	32

**Table 3:** Total reported events F: Fondaparinux, LMWH: Low molecular weight heparin, VTE: Venous thromboembolisms, DVT: Deep vein thrombosis, PE: Pulmonary embolism, M: Mortality, B: Bleeding

Study	VTE in LMWH patients (%)	VTE in F patients (%)	DVT in LMWH patients (%)	DVT in F patients (%)	PE in LMWH patients (%)	PE in F patients (%)	M in LMWH patients (%)	M in F patients (%)	B in LMWH patients (%)	B in F patients (%)
Cardillo et al., (2021) [11]	9 (0.09)	2 (0.02)	5 (0.05)	2 (0.02)	4 (0.04)	0 (0.0)	6 (0.06)	4 (0.04)	3 (0.03)	2 (0.02)
Russo et al., (2020) [12]	10 (0.08)	3 (0.025)	5 (0.04)	2 (0.016)	4 (0.03)	0 (0.0)	7 (0.06)	5 (0.04)	3 (0.025)	3 (0.025)

**Figure 5:**
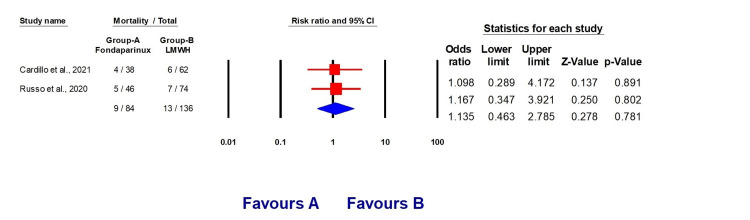
Forest plots explaining mortality rate with fondaparinux and LMWH LMWH: Low molecular weight heparin

**Figure 6:**
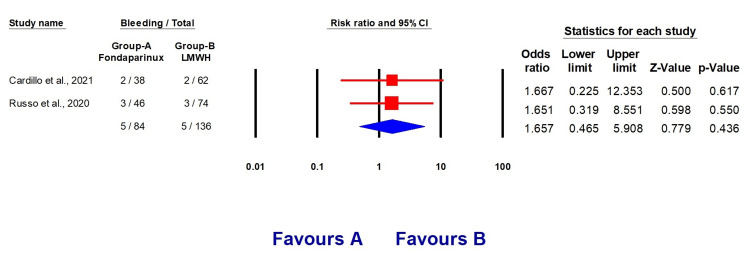
Forest plots explaining bleeding incidence with fondaparinux and LMWH LMWH: Low molecular weight heparin

However, the results indicate a reduced death rate in the LMWH groups (OR 1.135, 95% CI (0.463, 2.785), p = 0.781) and a decreased incidence of bleeding (OR 1.657, 95% CI (0.456, 5.908), p = 0.436) (Figures 5-6).

**Figure 7:**
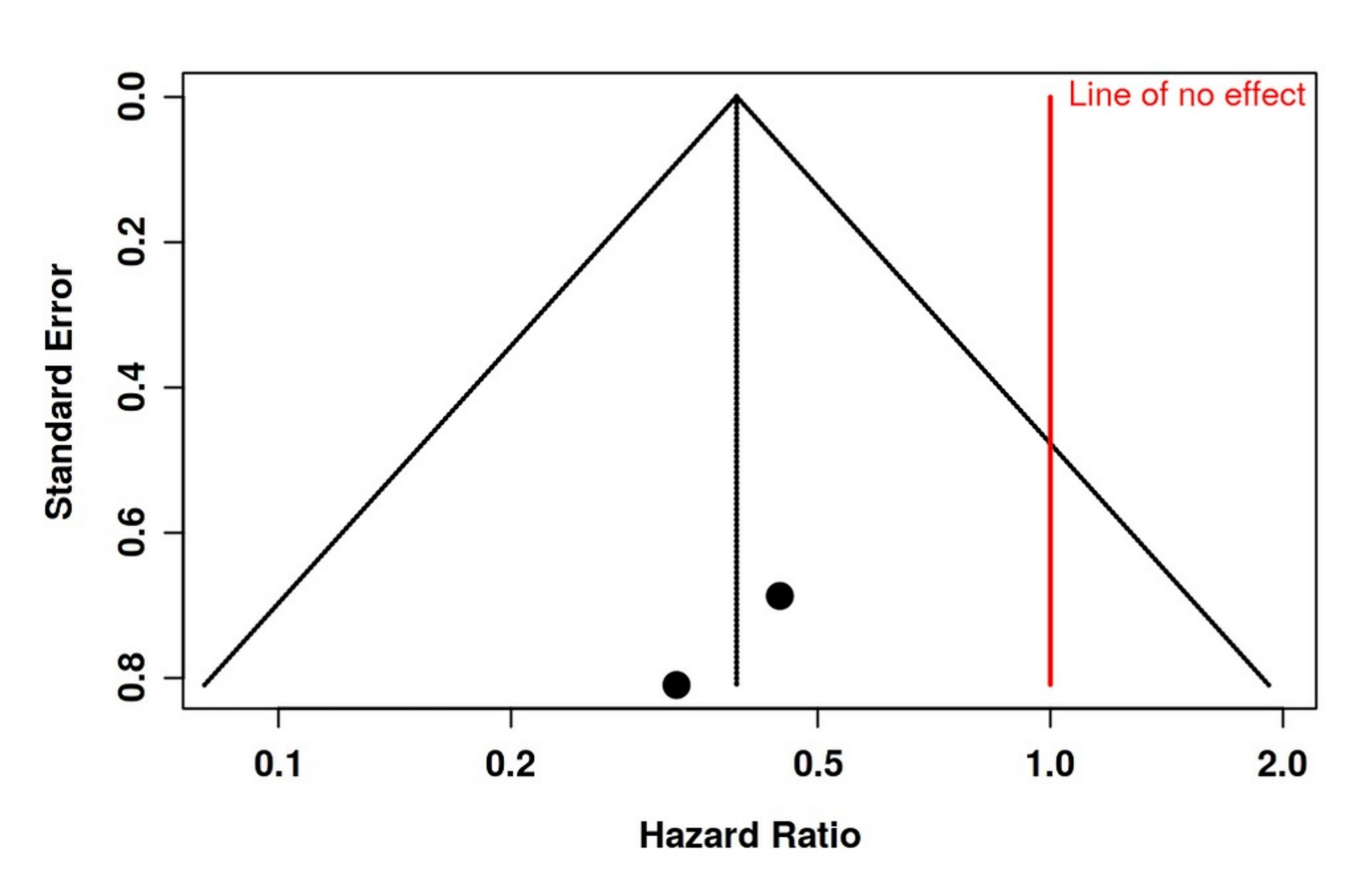
Funnel plot depicting no significant publication bias regarding venous thromboembolism development in the studies included

**Figure 8:**
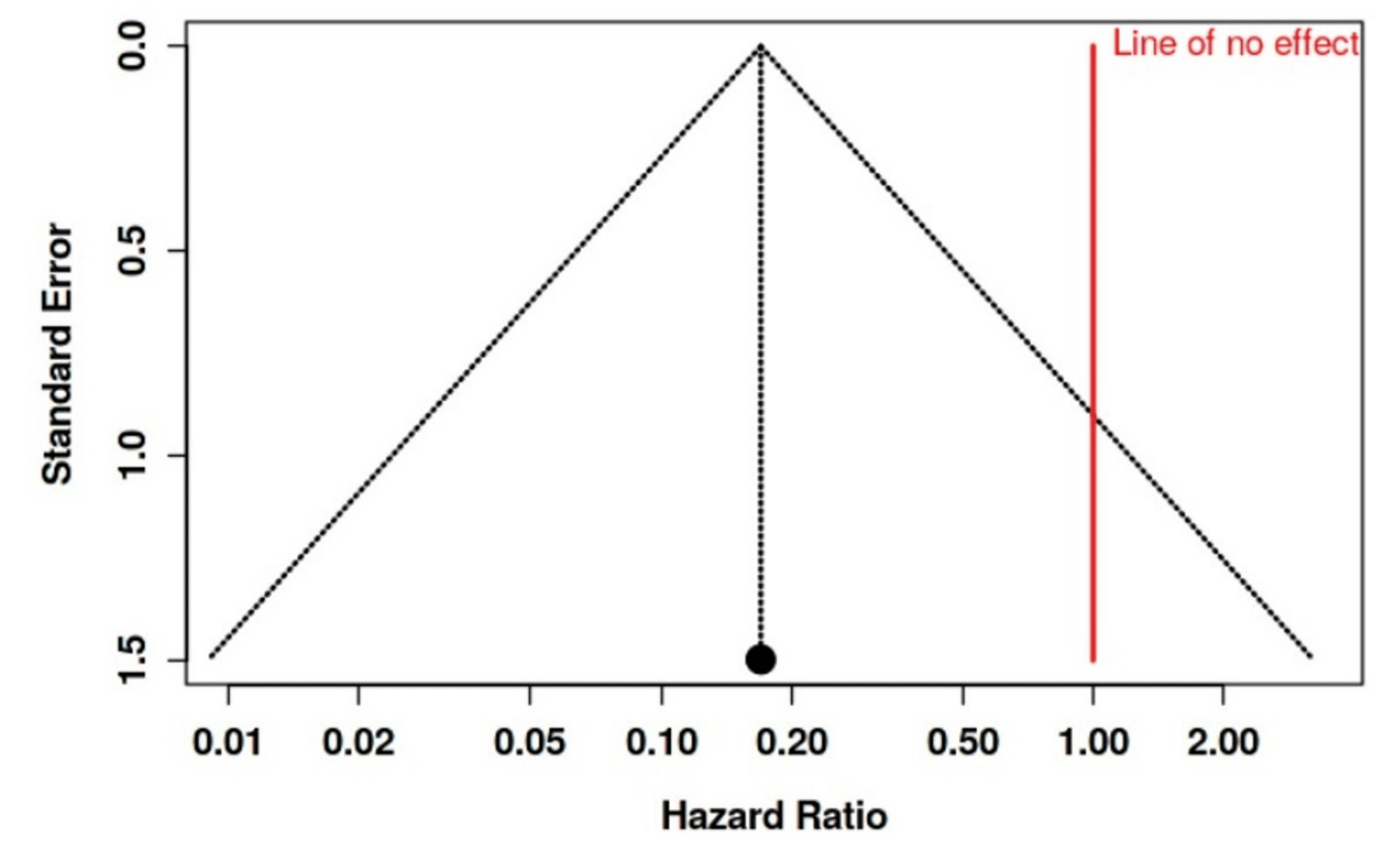
Funnel plot depicting no significant publication bias regarding pulmonary embolism development in the studies included

**Figure 9:**
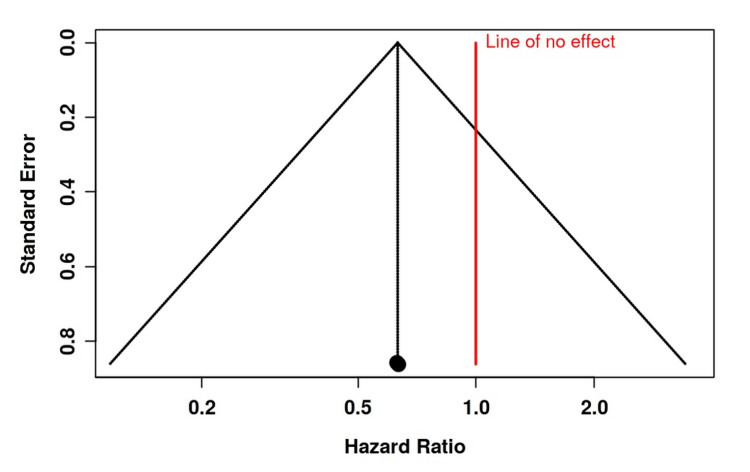
Funnel plot depicting no significant publication bias regarding deep venous thromboembolism development in the studies included

**Figure 10:**
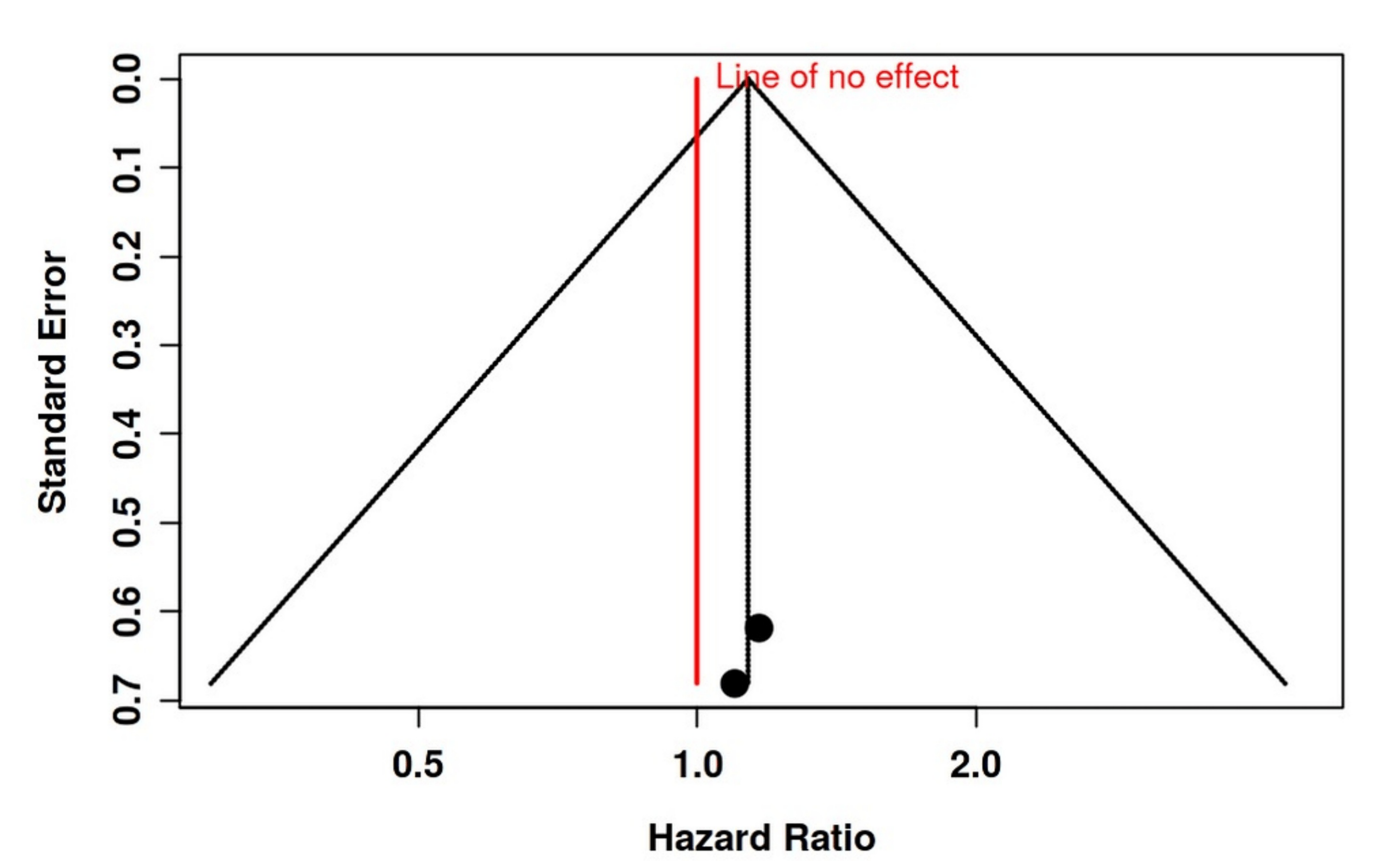
Funnel plot depicting no significant publication bias regarding mortality rate in the studies included

**Figure 11:**
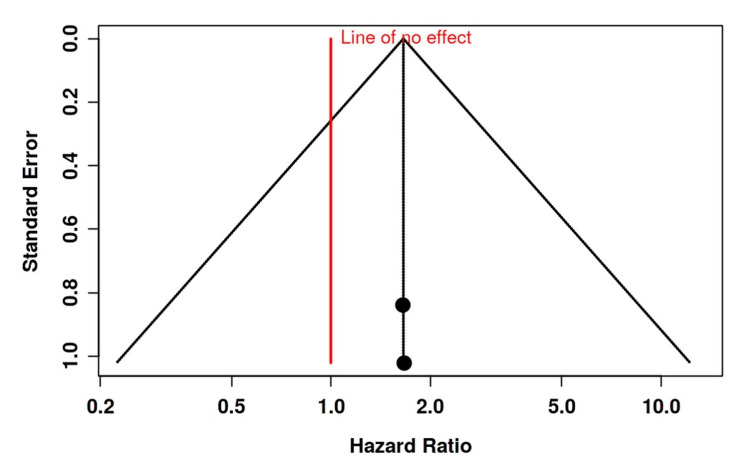
Funnel plot depicting no significant publication bias regarding bleeding incidence in the studies included

The funnel plots (Figures 7-11) show that the exposure to publication bias was not statistically significant.

Discussion

Numerous studies have demonstrated the elevated incidence of coagulopathy and venous thromboembolism (VTE) in hospitalized COVID-19 patients, yet there is little understanding of the possible correlation between antithrombotic treatments and the clinical manifestations or outcomes of COVID-19 [13]. The World Health Organization advocates utilizing heparin as a pharmaceutical prophylactic for preventing VTE in COVID-19 patients and recommends a once-daily dosage regimen instead of unfractionated heparin to minimize the need for personal protection equipment and risks to healthcare personnel [13].

The findings of the earlier study investigating the mortality risk among COVID-19 patients taking various dosages of anticoagulation are consistent with our findings, despite the inclusion of additional antithrombotic medications [14]. Notably, we presented evidence that the likelihood of experiencing any thrombotic events was consistently reduced in the cohorts of fondaparinux patients compared to LMWH patients. The observed phenomena suggest that anticoagulants, particularly medicines derived from heparin, may influence the progression of illness via processes that extend outside their primary effects. Significantly, investigations have shown that heparin has both anti-inflammatory and direct antiviral properties against SARS-CoV-2. Heparin competitively inhibits the viral entrance into the host cells by binding permanently to the spike protein. Nevertheless, LMWHs have revealed a reduced affinity for the spike protein and thus may have little or rather no direct antiviral effect [14,15].

The meta-analysis conducted by Kamel et al. [16] identified a positive correlation between inpatient anticoagulation and fatalities. Similarly, Wijaya et al. [17] observed a trend toward decreased mortality in patients who received both mechanical ventilation and pharmacological anticoagulation. The comprehensive review and meta-analysis undertaken by Parisi et al. [18] similarly found a correlation between anticoagulation and a reduced mortality rate. Although the previously mentioned meta-analysis included various kinds of antithrombotic drugs, including oral anticoagulants and unfractionated heparin, our objective was specifically to assess the efficacy and safety of LMWH/F.

The primary result of the current study was that there was no notable disparity in the development of bleeding episodes between COVID-19 patients receiving fondaparinux and those receiving LMWH thromboprophylaxis. However, LMWH demonstrated a greater overall therapeutic advantage in comparison to fondaparinux. Initial findings confirm that fondaparinux is safe and efficacious when compared to LMWH for COVID-19 patients admitted to internal medicine units.

The main strength of the present meta-analysis lies in its execution by a diverse team of experts using a rigorous methodological framework based on a pre-established PRISMA methodology. Furthermore, we used a comprehensive and rigorous literature search conducted by independent reviewers. The present study differs from earlier meta-analyses by specifically examining the effects of LMWH/F, the most frequently and extensively used anticoagulant in clinical practice. This is particularly relevant for hospitalized patients because LMWH and fondaparinux are characterized by their simplicity of use (once or twice daily) and minimal monitoring (in comparison to unfractionated heparin).

It is important to recognize the limitations of this research. First, the design specifically included only LMWH and excluded unfractionated heparin or direct oral anticoagulants. Secondly, there is a lack of adequate evidence on the potential hazards and benefits of administering these medications in mild cases that are treated in outpatient settings. Moreover, careful interpretation of our findings is necessary because of the average quality and the heterogeneity of the studies included.

## Conclusions

Both medications have shown antithrombotic effects in individuals with COVID-19. Fondaparinux showed little benefit in decreased occurrence of VTE events. These added indicators identified in our investigation should constantly be integrated with the clinical aspects. Based on a positive net clinical advantage over fondaparinux, this study confirms the safe use of LMWH for VTE prevention in hospitalized COVID-19 patients, regarding favorable bleeding and mortality outcomes.
